# AthRiboNC: an Arabidopsis database for ncRNAs with coding potential revealed from ribosome profiling

**DOI:** 10.1093/database/baae123

**Published:** 2024-12-17

**Authors:** Yi Shen, Liya Liu, Enyan Liu, Sida Li, Yuriy Orlov, Vladimir Ivanisenko, Ming Chen

**Affiliations:** Department of Bioinformatics, College of Life Sciences, Zhejiang University, Hangzhou 310058, China; Department of Bioinformatics, College of Life Sciences, Zhejiang University, Hangzhou 310058, China; Department of Bioinformatics, College of Life Sciences, Zhejiang University, Hangzhou 310058, China; Department of Bioinformatics, College of Life Sciences, Zhejiang University, Hangzhou 310058, China; Institute of Biodesign and Complex Systems Modeling, Sechenov First Moscow State Medical University (Sechenov University), Moscow 119991, Russia; Institute of Cytology and Genetics, Siberian Branch of Russian Academy of Sciences, Novosibirsk 630090, Russia; Department of Bioinformatics, College of Life Sciences, Zhejiang University, Hangzhou 310058, China

## Abstract

Non-coding RNAs (ncRNAs) are traditionally considered incapable of encoding proteins, but new evidence suggests that small open reading frames (sORFs) within ncRNAs can actually encode biologically functional small peptides. Despite growing recognition of their importance, a systematic exploration of plant ncRNAs with coding potential has remained largely uncharted territory, especially in the context of their translational activities. By collecting and analyzing Ribo-Seq data from 226 *Arabidopsis thaliana* samples, we have integrated extensive information on *Arabidopsis* ncRNAs with coding potential and developed the AthRiboNC database, a novel and dedicated database that consolidates extensive information on ncRNAs with coding potential in *Arabidopsis*. AthRiboNC covers detailed information on 2743 long non-coding RNAs, 255 microRNAs, and 1871 circular RNA in *Arabidopsis*, along with 40 162 ORFs identified from these ncRNAs. The database also constructs co-expression networks for ncRNAs with coding potential, revealing correlations and potential biological function interpretations. With a commitment to accessibility and ease-of-use, AthRiboNC features a clear and intuitive interface. We hope that AthRiboNC will serve as a valuable resource for exploring the coding potential of plant ncRNAs.

**Database URL**: https://bis.zju.edu.cn/athribonc

## Introduction

In traditional biological views, non-coding RNA (ncRNA) refers to RNA that either lacks significant coding capacity or has very low coding potential. Although ncRNAs do not directly code for proteins, they play complex roles in the regulation of gene expression within organisms Generally, ncRNAs can be categorized into three classes based on their length: small molecular RNAs (smRNAs, 18–30 bp in length), medium-sized ncRNAs (31–200 bp), and long non-coding RNAs (lncRNAs, longer than 200 bp). Small molecular RNAs include microRNAs (miRNAs) and small interfering RNAs (siRNAs). Medium-sized ncRNAs are primarily structural ncRNAs, such as transfer RNAs (tRNAs, approximately 75–87 bp in length) and small nucleolar RNAs (snoRNAs, around 150 bp) involved in ribosome synthesis [1]. The mechanisms and biological functions of lncRNAs are highly diverse; they regulate gene expression by influencing the stability of other RNAs or proteins and participate in processes such as dosage compensation, genomic imprinting, and X-chromosome inactivation [2]. Additionally, circular RNA (circRNA), as a special type of closed-loop RNA, typically originates from back-splicing events where the 3ʹ end of a pre-mRNA exon or intron connects with the upstream 5ʹ end, forming a covalently closed circular structure [3].

Recent studies have demonstrated that ncRNAs, previously thought to lack translational activity, can indeed be translated into functional amino acid sequences. For example, the primary transcripts of miRNAs (pri-miRNAs), lncRNAs, and circRNAs can encode biologically active peptides (non-coding peptides, ncPEPs) that often originate from short open reading frames within these ncRNAs [4]. In animals, a substantial amount of information on ncRNA-encoded peptides has been integrated and validated. The SPENCER database [5] has collated data on 29 526 ncPEPs across 15 different cancer types in humans, with 22 060 of these peptides having been experimentally verified in other studies. The ncPEPs are spotted to be involved in the regulation of various biological processes, such as the processes of tumor invasion and migration [6]. In plants, pri-miR171b from alfalfa (*Medicago truncatula*) and pri-miR165a from *Arabidopsis thaliana* encode peptides (mtr-miPEP171b and ath-miPEP165a, respectively), which enhance the accumulation of their corresponding mature miRNAs, leading to the negative regulation of target genes during root development [7]. The *Arabidopsis* small peptide miPEP858a derived from a miRNA controls flavonoid biosynthesis, influencing plant growth and development [8]. Identified plant endogenous ncRNA-encoded peptides often show potential in regulating plant physiological processes and even altering plant traits. The small open reading frames (sORFs) and their encoded peptides in these plants are theoretically predictable and characterizable in terms of their functions [9]. However, the number of plant ncRNA-encoded peptides that have been convincingly validated remains quite small.

Ribosome profiling, also known as Ribo-seq, is a high-throughput sequencing technology used to study the process of protein translation [10, 11], serving as a bridge between the transcriptome and the translatome. Ribo-seq uses a translation elongation inhibitor to freeze the translating ribosomes and performs deep sequencing on ribosome-protected mRNA fragments (RPFs), capturing the positions of ribosomes on mRNAs, which can reveal the dynamics of protein synthesis [12]. This technique provides genome-wide information on RNA translation, not limited to known coding sequences (CDS) but also capable of detecting potential translational activity in non-coding RNAs (ncRNAs) [13], offering a powerful tool for studying ncRNA-encoded peptides. Thus, we have carefully collected ribosome profiling sequencing data from 226 *A*. thaliana samples sourced from the GEO/SRA databases, which serve as the raw data foundation for our database. The aim is to identify translated ORFs and translation initiation sites (TIS) within ncRNAs of *A. thaliana*.

Despite substantial validation of the biological functions of non-coding RNA (ncRNA)-encoded peptides in animals, particularly humans and mice, our systematic understanding of plant-derived ncRNA-encoded peptides remains limited. For instance, the ncEP database [14] catalogs experimentally verified ncRNA-encoded peptides from 18 species, encompassing merely eight *A. thaliana* peptides, three soybean peptides, two alfalfa peptides, and one maize peptide. Furthermore, evidence for peptide coding by plant ncRNAs is often rather isolated, and we currently lack a comprehensive understanding of the regulation of the translational potential of these ncRNAs and the interactions among the resulting small peptides. To address this gap, we introduce a novel database dedicated to the coding potential of ncRNAs in *A. thaliana*, termed AthRiboNC. Our approach commenced with the processing of raw Ribosome Profiling (Ribo-seq) FASTQ files, enabling us to ascertain translation levels of distinct ncRNAs across various experimental treatments. This comprehensive analysis facilitates the elucidation of correlations and networks among *A. thaliana* ncRNA-encoded peptides, alongside their potential biological functions. Our ambition is that the AthRiboNC database will provide fresh insights into the coding potential of ncRNAs in this model plant, *A. thaliana*, and concurrently serve as an invaluable resource for probing the translational processes of plant ncRNAs.

## Materials and Methods

### Data sources

Our database is dedicated to *A. thaliana*, a model organism widely used in plant research. Unlike many other plant species, *Arabidopsis* benefits from extensive reference genome annotations, including non-coding RNAs like lncRNAs and miRNAs. However, it is important to note that there is a relative scarcity of available Ribo-seq data for most plants.

To address this gap, we conducted a search using keywords ‘Arabidopsis thaliana [orgn] AND Ribo-seq OR ribosome profiling’ within the NCBI GEO and SRA datasets. This meticulous process involved reviewing and evaluating data for availability and reliability, ultimately leading to the acquisition of 226 samples from 26 distinct BioProjects. Each dataset and its corresponding original literature were manually examined to ensure data integrity and to document the experimental metadata, including plant tissue types, ecotypes, and any treatments applied to the biological samples.

We obtained the *Arabidopsis* TAIR10 reference genome sequence and genome annotation files [15] from Ensembl Plant. On account that circular RNA (circRNA) arises from extensive alternative back-splicing and is not annotated in the reference genome, we compiled a library of *A. thaliana* circRNAs from PlantcircBase [16]. This database includes over 50 000 circRNA transcripts of *A. thaliana*, predicted through various methods.

### Data preprocessing

After converting SRA files into sequencing raw data in FASTQ format using the SRAToolkit, we employed Cutadapt [17] to automatically identify and excise all data adapters and utilized Trimmomatic [18] for base trimming of low-quality segments, concurrently discarding reads of substandard quality. More precisely, we executed trimming of bases at both ends and within sliding windows that fell below a predetermined quality threshold. Additionally, we adjusted the length of the reads to span between 25 and 34 nucleotides, aligning with the typical length of ribosome-protected RNA fragments. The integrity of the preprocessed data was meticulously evaluated using FastQC to confirm their reliability.

### Alignment and quantification

After preprocessing, we assessed the translational levels of reference genes and circRNAs through three alignment steps using HISAT [19].

#### Step One

The preprocessed data were aligned to the tRNA and rRNA library in the TAIR10 reference genome. Reads that did not align were retained, removing the influence of rRNA and tRNA in ribosome profiling.

#### Step two

The retained reads were aligned to the TAIR10 reference genome to measure the translational levels of canonical genes (including lncRNAs and miRNAs) in the Ribo-seq data.

#### Step three

Reads that did not align to the reference genome were then aligned to the *Arabidopsis* circRNA library from PlantCircBase.

To minimize false positives, samtools [20] was used to remove secondary and reverse alignments in **Steps Two** and **Three**. Specifically, a maximum of two mismatches per read was allowed in **Step Two**, while no mismatches were permitted in **Step Three** due to the large circRNA library containing 52 393 entries, which could increase false positive rates.

FeatureCounts [21] was used to quantify the BAM files from the alignment, providing raw counts of gene translational levels in a sample-by-gene matrix. The raw count matrices from **Step Two** (reference genes) and **Step Three** (circRNAs) were merged, and TPM (transcripts per million) was calculated using the formula:


$$TPM = \frac{{{\mathrm{count}}\left( i \right)/{\mathrm{length}}\left( i \right)}}{{\mathop \sum \nolimits{\left( {{\mathrm{count}}/{\mathrm{length}}} \right)}}}$$

In this formula, the length of the reference gene is the effective gene length calculated by FeatureCounts based on all transcripts of the gene. For circRNAs, non-back-splicing sites are aligned to the reference genome in **Step Two**, while the portions aligned to the circRNA library in **Step Three** correspond to back-splicing junctions. To avoid underestimating the effective translational levels of circRNAs, an average circRNA length of 30 was used, and the effective gene length for circRNAs was considered to be 60.

### Identification of sORFs

We use ORFFinder [22] to search for potential small open reading frames (sORFs) within the transcript sequences of non-coding RNAs. The running parameters are configured with a minimum sORF length of 30 amino acids, and both ATG and non-ATG start codons are enabled. To reduce the likelihood of false positives, sORFs shorter than 30 amino acids are disregarded during prediction. However, this does not imply that sORFs shorter than 30 amino acids are entirely non-existent. It is important to note that ORFFinder predicts sORFs based solely on nucleotide sequences, without incorporating actual expression data from Ribo-seq.

To achieve a more accurate identification of sORFs undergoing translation in Ribo-seq data, we employ the predictive capabilities of Ribo-TISH [23]. For this analysis, we use BAM files aligned to the TAIR10 reference genome and the corresponding GTF annotation file as input for Ribo-TISH, which then outputs predicted sORFs with confidence levels derived from reference transcripts. Both ATG and non-ATG start codons are enabled. Since circRNAs are not annotated in the TAIR10 reference genome, our analysis focuses solely on miRNAs and lncRNAs in this case.

### Co-expression network analysis

The sample-by-gene TPM expression matrix was subjected to co-expression network analysis using the WGCNA package [24] in R. Lowly expressed coding genes and non-expressed non-coding genes in the Ribo-seq data were excluded. The filtered expression matrix includes 33 869 genes across 226 samples.

Following the general WGCNA analysis pipeline, adjacency and TOM similarity between genes were calculated based on the Pearson correlation matrix, with an optimal soft-thresholding power of eight selected for this analysis. Using the computed TOM similarity matrix, a co-expression network was constructed through the one-step method. All genes were allocated into 24 different modules, while MEgrey represents genes that could not be classified into any other modules.

The sample’s categorical experimental data—comprising age, heat stress, dark stress, chemical stress, microbial stress, flood stress, and plant hormone treatments—were manually converted into numerical values to form the phenotype matrix. Pearson correlations were calculated between genes and phenotypes, as well as between samples and phenotypes. Genes with gene-sample membership >0.9 and gene-phenotype correlation >0.25 in each module were considered hub genes of that module. Modules containing more than 10 hub genes underwent enrichment analyses for KEGG pathways and Gene Ontology (GO) terms using the clusterProfiler package [25] in R. This approach provides a comprehensive understanding of the biological processes, cellular components, and molecular functions associated with these key genes, as well as their potential roles in specific metabolic or signaling pathways. Non-coding RNAs (ncRNAs) showing a high correlation with modules may be crucial in regulating the biological functions associated with these modules. Their expression and translational levels could indicate their role in modulating the module’s activity, potentially influencing regulatory networks or being targets of regulation within the module’s biological processes.

### Database construction

**Figure 1.**
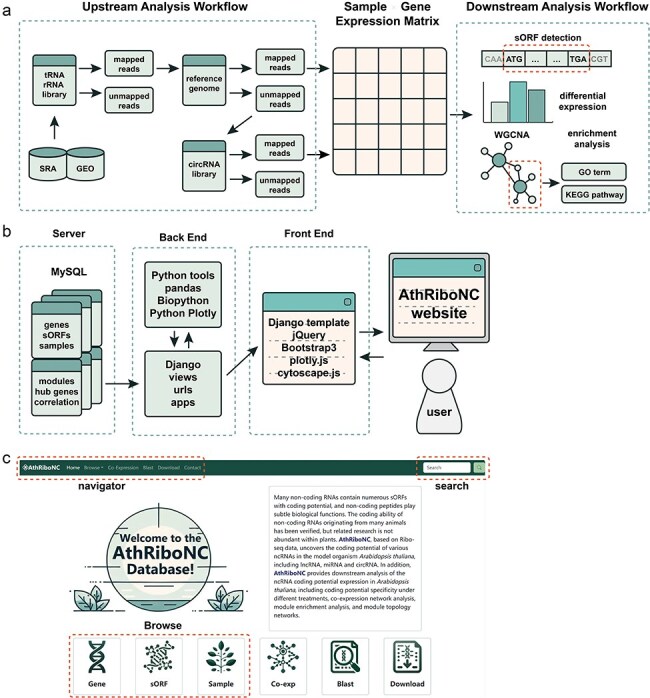
The construction workflow of AthRiboNC database. (a) The basic workflow for data analysis and processing in AthRiboNC. (b). The basic framework for building the AthRiboNC website. (c) The homepage showcases the main functional modules of AthRiboNC database.

We developed the backend of the database website using Django, a Python-based web framework. For the frontend, database templates were designed with HTML and CSS, and jQuery was used to enhance user interaction. Interactive visualization charts were created with Python Plotly and the R package heatmaply. Collected data is stored in MySQL, and the entire database is hosted in a Docker container running on an Ubuntu 20.04 Linux server (Fig. 1a, b). The database is currently accessible at https://bis.zju.edu.cn/athribonc/.

## Results

### Database description

Athribonc offers a comprehensive repository of *Arabidopsis* ncRNAs with translational potential, derived from Ribo-seq data. The database includes 4869 *Arabidopsis* ncRNAs with effective expression in Ribo-seq data, comprising 2743 lncRNAs, 255 miRNAs, and 1871 circRNAs. Using ORFFinder based on sequence features, 31 928 sORFs were identified within these ncRNAs. Additionally, 8234 sORFs were detected using Ribo-TISH, which considers both sequence features and actual translational levels. Athribonc features a user-friendly interface, allowing easy access to functional modules including Browse, Co-Expression, Blast, and Download from the homepage and navigation bar (Fig. 1c).

### Browsing ncRNA Genes, sORFs and Ribo-seq Samples

The Browse page of Athribonc provides essential information about *Arabidopsis* Ribo-seq experiments and ncRNAs with coding potential. It includes three sub-pages: Gene, sORFs, and Sample. The Gene Page lists ncRNAs with coding potential, allowing users to sort or filter entries by various criteria such as ncRNA gene type, genomic location, and Ribo-seq expression. The sORFs Page details sORFs predicted by ORFFinder or Ribo-TISH within these coding potential ncRNAs, enabling users to view sORF sequences and sort or filter entries by attributes like ncRNA gene type, start codon, source, and sORF length.

In our database, miRNAs are named according to the nomenclature from miRBase [26], circRNAs use IDs from PlantcircBase, and lncRNAs follow the TAIR Gene Model Naming Convention ID corresponding to the reference gene of the ncRNA transcript. The parent gene is referenced by the TAIR Gene Model Naming Convention ID for miRNAs and lncRNAs. For circRNAs, which originate from back-splicing, the parent gene ID is sourced from PlantcircBase. For ncRNA transcripts, miRNA and lncRNA transcripts use TAIR Gene Model Naming Convention IDs, while circRNA transcripts use IDs from PlantcircBase. The Sample Page provides all collected Ribo-seq sample data, including Tissue, Ecotype, Age, and Experimental Treatments, which have been manually curated. Users can also follow hyperlinks to navigate to the NCBI SRA database or Bio Project page.

**Figure 2.**
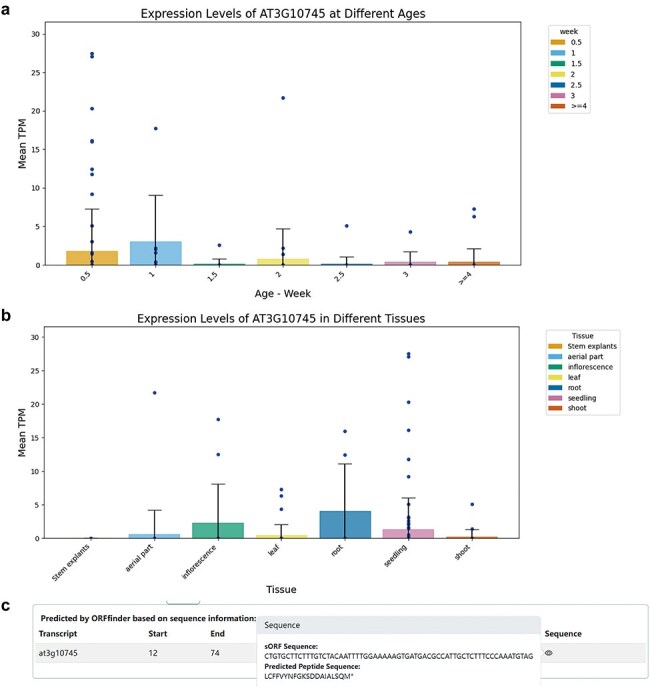
Information on ncRNAs with coding potential. (a) Expression levels of the miRNA gene AT3G10745 (MIR158a) in different Ribo-Seq samples across different ages. (b) Expression levels of the miRNA gene AT3G10745 (MIR158a) in different Ribo-Seq samples across different tissues. (c) ORFfinder-identified sORF with coding potential in the primary transcript (pri-miRNA) of AT3G10745 and corresponding predicted small peptide.

Within the Gene and sORF sub-interfaces, users can click to access detailed entry pages for each gene. These entries include a Gene Summary with the ncRNA gene’s name, genomic location, and nucleotide sequence. The corresponding genomic locations and annotations were visualized using JBrowse [27]. Transcript Information lists all transcripts under the ncRNA gene, noting that a single lncRNA gene may have multiple transcripts due to alternative splicing; and sORF Information lists all predicted coding potential sORFs and their sequences. The Expression section visually represents the translational-level specificity of the ncRNA across different tissues and age samples using bar charts, while Co-Expression displays genes most highly correlated with the expression of the ncRNA, including both coding and non-coding genes (Fig. 2).

Furthermore, a search function on the right side of the database navigation bar allows users to quickly locate entries of interest within the Browse section. This function supports string matching based on ncRNA name, parent gene name, SRA Run accession, Bio Project, or Sample Name for searching experimental sample information.

### Co-expression network

**Figure 3.**
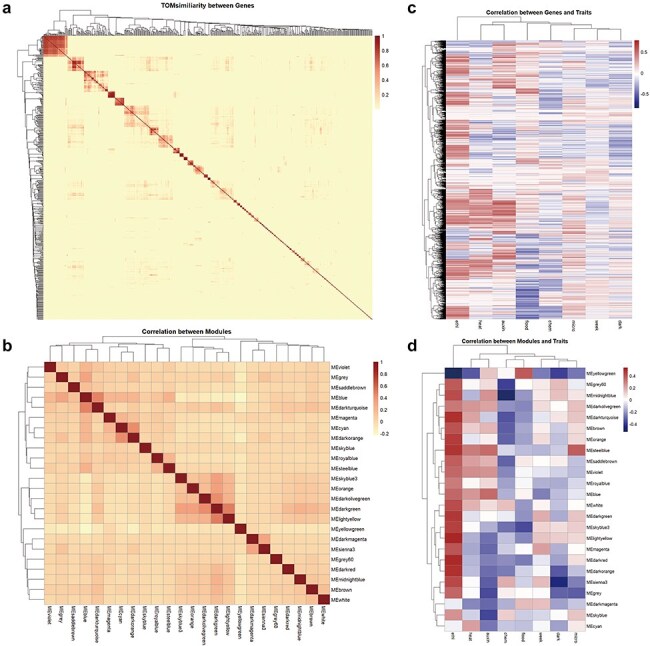
Correlation of genes, co-expression network modules, and traits. (a) Visualized heatmap of the TOMsimiliarity between ncRNA genes with coding potential. (b) Visualized heatmap of the correlation between 24 modules. (c) Visualized heatmap of the correlation between genes and traits. (d) Visualized heatmap of the correlation between module eigengenes and traits.

On the Co-expression page, we present correlation heatmaps for module × module, module × trait, and hub genes within modules × hub genes derived from Ribo-seq data. This feature allows users to quickly and intuitively identify co-expression network modules related to traits of interest. Although empirical evidence for the interaction of peptides encoded by *Arabidopsis* ncRNAs is currently lacking, we can infer their potential roles in biological processes from the co-expression patterns of ncRNAs with coding potential observed in Ribo-seq data (Fig. 3).

**Figure 4.**
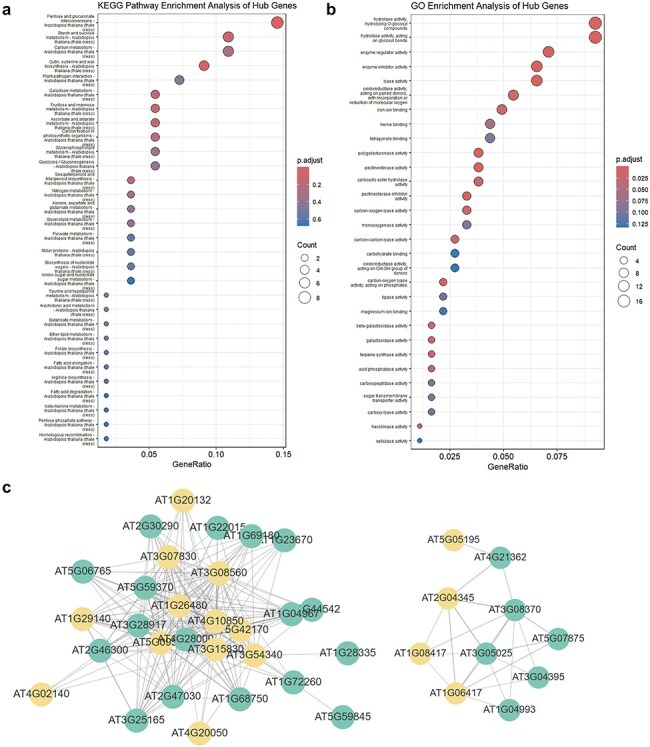
WGCNA module enrichment analysis results and visualized network topology. (a) KEGG pathway enrichment analysis results for hub genes in the MEdarkturquoise module. (b) GO enrichment analysis results for hub genes in the MEdarkturquoise module. (c) Visualization of the network correlation strength between several genes in the MEdarkturquoise module using Cytoscape. Orange nodes represent hub genes, while green nodes represent non-hub genes.

This section includes entries for all coding potential ncRNA genes featured in the Browse section, as well as protein-coding genes identified as hub genes within modules. Each gene entry displays its type, the module it belongs to, its status as a hub gene within the module, and its module membership. We have identified 65 ncRNAs, including 2 miRNAs, 40 lncRNAs, and 23 circRNAs, that serve as hub genes within modules. These ncRNAs with coding potential exhibit high module membership and trait association, suggesting their coding potential may be significantly related to aging or environmental stress. Users can click the arrow next to each module to access a detailed page for that module. Meanwhile, for coding genes, we also provided hyperlinks to The Arabidopsis Information Resource. Each module’s page lists the number and proportion of various coding potential ncRNAs within the module. Additionally, we have randomly selected 50 genes to create a TOM similarity heatmap, which visually represents the topological structure of the gene interaction network within the module, reflecting interaction strength and gene clustering. For modules with more than 10 hub genes, the Enrichment Analysis section provides results from KEGG pathway and GO term enrichment analyses for the hub gene set. The Correlation Network section visualizes the co-expression network of selected genes within the module using Cytoscape [28], illustrating hub gene density and interaction strength (Fig. 4).

### Blast tool

In the BLAST section of Athribonc, we have implemented an online BLAST function using the blastn and blastp tools from the Biopython package. This feature allows users to align submitted nucleotide or amino acid sequences against all available coding potential non-coding RNA (ncRNA) genomic regions, transcripts, or small open reading frames (sORFs) in our Arabidopsis database. This functionality aids in determining whether the target sequence possesses coding potential. For example, three small peptide sequences from *A. thaliana*, with OpenProt IDs IP_4275268, IP_4275229, and IP_4330112, are listed in the OpenProt database [29] with an MS score and can be correctly aligned to sORFs sequences in AthRiboNC.

### Data download page

On the Download page, users can access and download the contents of our database. In the Meta Information section, users can download visible data from the Gene, sORF, and Sample sections on the Browse page, as well as all sample × gene expression matrices saved as RData files. In the Sequence section, we offer sequences of genomic regions, transcripts, and sORFs, which are also used as the database for BLAST. The Analysis Results section provides the outcomes of WGCNA analysis, including module assignments for each gene and the results of KEGG pathway enrichment and GO enrichment analyses for each module.

### Case study

Using the OpenProt database entry IP_4332194 as an example, which is classified as an Isoform Protein with an MS score, we aim to explore the origin and potential function of this protein. We aligned its amino acid sequence, MNSGLCLETSLFDFLCKYHEMPMDRFFQAI, against the sORF BLASTp database on the BLAST page with the default E-value of 1e-5. The alignment showed that IP_4332194 correctly aligns with the AT3G29644 gene. Further investigation into the AT3G29644 gene entry revealed that it has five alternatively spliced lncRNA transcripts and a rich presence of sORFs. Upon excluding zero expression data, we observed that AT3G29644 is expressed in *Arabidopsis* Ribo-seq samples across various ages and tissues, with notably higher translation levels in the inflorescence and shoot. In the Co-expression interface, we found that AT3G29644 is associated with the MEblue module. The Ribo-seq expression profile of MEblue is negatively correlated with aging and positively correlated with the application of ethylene and auxin. Enrichment analysis indicates that the biological functions enriched in MEblue include respiration, amino acid synthesis, protein synthesis and modification, and protein degradation. Based on the super-resolution ribosome profiling using data analysis workflow RiboTaper [30], an sORF in the AT3G29644 gene was indeed identified, with translation occurring in the shoot [31]. Additionally, studies suggest that the expression of the AT3G29644 gene is regulated by SDE3, which may be related to antiviral defense or gene silencing, and could also be involved in the silencing of transposable elements or newly acquired genomic regions [32].

## Discussion

Here, we have developed a database for *A. thaliana* non-coding RNAs (ncRNAs) with coding potential, named AthRiboNC. This database features several modules: Browse, Co-expression, Blast, and Download. Researchers can access information on *Arabidopsis* ncRNAs with coding potential, including their genomic locations and sequence details. By analyzing the expression levels of ncRNAs in Ribo-Seq data across various tissues and developmental stages, scientists can infer the tissue-specific and developmental stage-specific coding potential of *Arabidopsis* ncRNAs. Additionally, we have constructed co-expression networks based on translation levels from diverse samples, enabling users to explore gene modules, correlations between modules and traits, the topological characteristics of the modules, and the enriched functions of module hub genes.

In summary, AthRiboNC offers a comprehensive integration of *Arabidopsis* Ribo-Seq data, providing detailed information about ncRNAs with coding potential. Users can perform BLAST alignments with nucleotide or amino acid sequences within our database, search for entries by gene or sample name, and filter results in the Browse interface according to genomic location, gene type, expression level, and other criteria. Our database is designed to be clear, user-friendly, and freely accessible.

Currently, AthRiboNC focuses exclusively on Ribo-Seq data specific to *A. thaliana*, as ribosome profiling sequencing data and genome annotations for ncRNAs are less abundant in other plant species. Furthermore, the data in AthRiboNC are exclusively derived from bulk Ribo-Seq data, providing translation information at the tissue level rather than at single-cell or spatial scales. Our goal is to accumulate more diverse, multi-species, and multi-scale Ribo-Seq datasets to offer broader insights into the translational potential of plant ncRNAs. We aim to continuously expand our database to enhance the understanding of the functions of plant ncRNAs and their encoded peptides, providing valuable data support for the design and validation of future experimental research.

## Data Availability

AthRiboNC is available at https://bis.zju.edu.cn/athribonc/. The upstream and downstream data is free to fetch in the Download page.
